# Remarks on the Increase of Medical Journals

**Published:** 1824-03-01

**Authors:** 


### Quarterly Periscope

OF

### PRACTICAL MEDICINE;

BEING THE

### Spirit of the Public Journals,

FOREIGN AND DOMESTIC.

*Paucis libris immorari et innutriri oportet, si velis aliquid trahere, quod in animo fideliter hæreat.* SENECA.

Duo vitia vitanda sunt in cognitionis et scientiæ studio. ***** Alterum est vitium, quod quidam nimis magnam operam conferunt in res obscuras atque difficiles, easdemque non necessarias. CICERO.

It is now nearly six years since we first projected the PERISCOPE, a department that has proved extremely useful, and has been adopted, to a greater or less extent, and under various denominations, by many of our cotemporaries in this and foreign countries. If it was a useful feature in our Journal then, it is indispensibly necessary now, when periodicals have multiplied at least four-fold in the short space of half a dozen of years.* It is now utterly impossible for any individual, or even society of individuals to take in, peruse, or even see, one half, or one fourth of the British periodical journals and transactions, which roll, like a torrent, from the press. A time has come when each individual and society must make their selection of one or more journals, trusting to these for some knowledge of what is going forward in the countless host of others, foreign and domestic.

As the present number of our Journal concludes the fourth volume, and as the next number (while by means of a spare title page it may form a continuation of the present series for all those who hold it)

* When we commenced the Quarterly Medico-Chirurgical Journal, in 1818, there were but *two Medical Journals* then in London. There are now a dozen, if not more. Some of them, though regularly published, we have not yet been able to see, excepting through the newspapers. We shall here endeavour to give a list of them, with their prices. *Quarterly.*—The Quarterly Journal of Foreign Medicine, &c. by Anderson, 4s. 6d. The Med. Chir. Review, 6s. *Monthly.*—The London Medical and Physical Journal, 2s. 6d. The London Med. Repository, 2s. 6d. The Medical Intelligencer, 1s. 6d. The Journal of Public Health, 1s. The Oracle of Health, 1s. The Gazette of Health, 1s. *Weekly.*—The Lancet, 6d. The Medico-Chirurgical and Philosophical Magazine, 4d. The Scalpel, 4d. The Medical Adviser, 3d. Several others, we understand are ready to appear!

will be the first of a new series, for the accommodation of new subscribers, we are desirous of drawing the attention of our readers to the future plan and execution of our *Periscope department*, as exemplified in the specimen presented them this quarter. We think that the condensed mass of practical and curious matter which we are enabled to dispense by means of the present construction of the Periscope, will merit approbation of our labours, and tend not a little to the benefit of our brethren, both at home and abroad. We shall say no more—but leave the result to the impartial examination and verdict of the public.

☞ We may be permitted to state that although we think there is some advantage to be derived from classing our subjects under the heads Pathology, Physiology, Therapeutics, &c. yet we do not consider it as of any consequence which should have the precedence. We merely place Pathology at the head; as one of the most important of our investigations in the present state of medical science.

